# Biological roles of RNA m^5^C modification and its implications in Cancer immunotherapy

**DOI:** 10.1186/s40364-022-00362-8

**Published:** 2022-04-01

**Authors:** Hang Song, Jianye Zhang, Bin Liu, Jing Xu, Biao Cai, Hai Yang, Julia Straube, Xiyong Yu, Teng Ma

**Affiliations:** 1 Department of Biochemistry and Molecular Biology, School of Integrated Chinese and Western Medicine, Anhui University of Chinese Medicine, Hefei, 230012 China; 2 Anhui Province Key Laboratory of Chinese Medicinal Formula, Hefei, 230012 China; 3 Key Laboratory of Molecular Target & Clinical Pharmacology and the State Key Laboratory of Respiratory Disease, School of Pharmaceutical Sciences & the Fifth Affiliated Hospital, Guangzhou Medical University, Guangzhou, 511436 China; 4 Department of Cellular and Molecular Biology, Beijing Chest Hospital, Capital Medical University/Beijing Tuberculosis and Thoracic Tumor Research Institute, Beijing, 101149 China; 5 Division of Surgical Research, Department of Surgery, University Medical Center Erlangen, Friedrich-Alexander University of Erlangen-Nuremberg, 91054 Erlangen, Germany; 6 Division of Molecular and Experimental Surgery, Department of Surgery, University Medical Center Erlangen, Friedrich-Alexander University of Erlangen-Nuremberg, 91054 Erlangen, Germany

**Keywords:** Epigenetics, RNA methylation modification, 5-methylcytosine (m^5^C), Non-coding RNAs, cancer immunotherapy

## Abstract

Epigenetics including DNA and RNA modifications have always been the hotspot field of life sciences in the post-genome era. Since the first mapping of N6-methyladenosine (m^6^A) and the discovery of its widespread presence in mRNA, there are at least 160-170 RNA modifications have been discovered. These methylations occur in different RNA types, and their distribution is species-specific. 5-methylcytosine (m^5^C) has been found in mRNA, rRNA and tRNA of representative organisms from all kinds of species. As reversible epigenetic modifications, m^5^C modifications of RNA affect the fate of the modified RNA molecules and play important roles in various biological processes including RNA stability control, protein synthesis, and transcriptional regulation. Furthermore, accumulative evidence also implicates the role of RNA m^5^C in tumorigenesis. Here, we review the latest progresses in the biological roles of m^5^C modifications and how it is regulated by corresponding “writers”, “readers” and “erasers” proteins, as well as the potential molecular mechanism in tumorigenesis and cancer immunotherapy.

## Overview of RNA 5-methylcytosine (m^5^C) modification

**Fig. 1:**
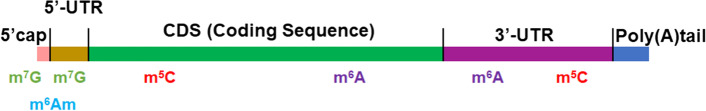
The distribution of methylation in mRNA. The preferential locations of each methylation within mRNA are shown

DNA and RNA modifications play crucial roles in various biological processes [1]. Compared with limited modifications detected in DNA, more than 170 modifications have been discovered in RNA including N6-methyladenosine (m^6^A), 5-methylcytosine (m^5^C) and 7-methylguanylate (m7G) (Fig. 1). They increase the complexity of the RNA species by acting on the tertiary structure, biogenesis, localization and function of RNA [2–4]. Among these modifications, mRNA N6-methyladenosine (m^6^A) is well acknowledged to be involved in translation, miRNA biosynthesis and gene attenuation [5, 6] (Fig. 1).

The discovery of DNA 5-Methylcytosine (m^5^C) can be traced back to the 1950s, earlier than the discovery of the double helix structure of DNA [7, 8]. In the 1970s, researchers discovered the m^5^C modification. Similar to DNA m^5^C [9], an active methyl-group from the donor, usually *S*-adenosyl-methionine (SAM), is added to the carbon-5 position of the cytosine base in RNA to form the m^5^C modification, which is likewise a widespread RNA modification detected in messenger RNA (mRNA) and non-coding RNAs including transfer RNA (tRNA), ribosomal RNA (rRNA), long non-coding RNA (lncRNA), small nuclear RNA (snRNA), micro RNA (miRNA) and enhancer RNA (eRNA) [10]. The m^5^C modifications have been reported in many species but its distribution seems to differ. For example, eukaryotic tRNA and mRNA have more m^5^C modification than bacterial mRNA and tRNA [9, 11, 12].

m^5^C modifications on tRNA and rRNA were extensively studied. In tRNA, m^5^C has been shown to maintain homeostasis, optimize codon–anticodon pairing, regulate stress response, and control translation efficiency and accuracy [13–18]. rRNA m^5^C modification is involved in glioma sensitivity to bioactive substrates of the stress-related enzyme NQO1 and structural stability of the tertiary rRNA–tRNA–mRNA complex under stress [19]. Until in the 1970s, researchers discovered the first m^5^C modification of eukaryotic mRNA, such as hamster cell mRNA and certain viral RNA but not SV40 RNA [20, 21]. Later, with the bisulfite sequencing in the whole transcripts of HeLa, a wide range of m^5^C modifications in mRNA and ncRNA were discovered. To date, mRNA m^5^C modifications have been associated with various biological processes, such as mRNA stability, splicing and nuclear–cytoplasmic shuttling [22–24]; DNA damage repair [25]; proliferation, and migration [26]; development, differentiation, and reprogramming of stem cells [27, 28]. Aberrant mRNA m^5^C modifications have been associated with the etiology of multiple diseases, including arteriosclerosis [29], autoimmune diseases [30] and cancer [31].

## 5-methylcytosine (m^5^C) modification in mRNA

With the development of high-throughput sequencing technology and the improvement of liquid chromatography sensitivity, the methods for the identification of the overall level of RNA methylation have been greatly developed. The current technical means used for the identification of the overall level of RNA methylation is mainly liquid chromatography coupled with MS (LC-MS). LC-MS/MS uses tandem mass spectrometry based on liquid-phase mass spectrometry, which is able to obtain both molecular and fragment ion peaks, allowing both qualitative and quantitative analysis of bases [32]. In contrast, bisulfite sequencing is a method that uses methylation to analyze different regions of DNA. In bisulfite sequencing, Bisulfite treatment can convert the unmethylated C bases in the genome into U, which becomes T after PCR amplification to distinguish them from the original C bases with methylation modifications, and then combined with high-throughput sequencing technology, a genome-wide DNA methylation map with single-base resolution can be drawn [33]. Moreover, a novel species-specific computational approach, Staem5, to accurately predict RNA m^5^C sites in *Mus musculus* and *Arabidopsis thaliana* was recently developed [34]*.*

Bisulfite sequencing shows that m^5^C modification is another abundant mRNA modification and may be another RNA epigenetic marker [35]. Recently, enhanced liquid chromatography-mass spectrometry (LC-MS) has shown that eukaryotes do show methylation and hydroxymethylation of endocytosine. Due to the loss of information about the location of m^5^C modification in LC-MS, the adaptability of bisulfite sequencing with RNA opens a new possibility of mapping m^5^C with nucleotide resolution in RNA. Using this method, Lukas Trixl and Alexandra Lusser found ~ 10,000 sites showing > 20% methylation and mapping to ~ 8500 mRNAs resulting in a rate of 0.43% m^5^C of all sequenced Cs, and they also published the first cytosine methylome for human cells [36].

Recently, Amort et al. detected about 7500 m^5^C sites (> 20% methylation) corresponding to 1650 mRNAs in mouse embryonic stem cells and 2075 m^5^C sites corresponding to 486 mRNAs in mouse brain. Their concluded that m^5^Cs modification mainly exist in the coding region and are enriched around the translation initiation site [37]. Another study also found that m^5^Cs modification localized to the untranslated regions (UTRs) of mRNA transcripts [38].

Another recently published article on HeLa cells and mouse cytosine, a matrix, identified about 3600 loci in about 2000 genes in HeLa cells and 2500-4400 sites (1000-1655 genes) in different mouse tissues [37, 39]. About 100 m^5^C sites in *Arabidopsis thaliana* mRNA were detected by bisulfite sequencing [40], while another study used meRIP-seq and found that 6045 peaks correspond to 4465 expressed genes [41]. MeRIP-seq was also used to detect the level of m^5^C in the archaebacterium *Sulfolobus solfataricus* and budding yeast, and a single site in yeast and 14 m^5^C modified mRNAs in S. solfataricus [12] were discovered respectively.

However, the exploration of the function of m^5^C in mRNA is not comprehensive and thorough, while there are some interesting findings, such as Yang et al. demonstrated that during the maternal-to-zygotic transition (MZT) of zebrafish, RNA m^5^C modification regulates maternal mRNA stabilization, highlighting the key role of m^5^C mRNA modification in early development [23]. More efforts are needed to uncover the m^5^C pandora-box roles besides in regulating mRNA stability.

## m^5^C modification in ncRNAs

It was found that m^5^C not only plays a role in regulating the stability of mRNA, but also regulating the stability of rRNA and tRNA. A large number of studies have reported that m^5^C modification is vital for translation regulation of tRNA and rRNA [42, 43].

**Fig. 2:**
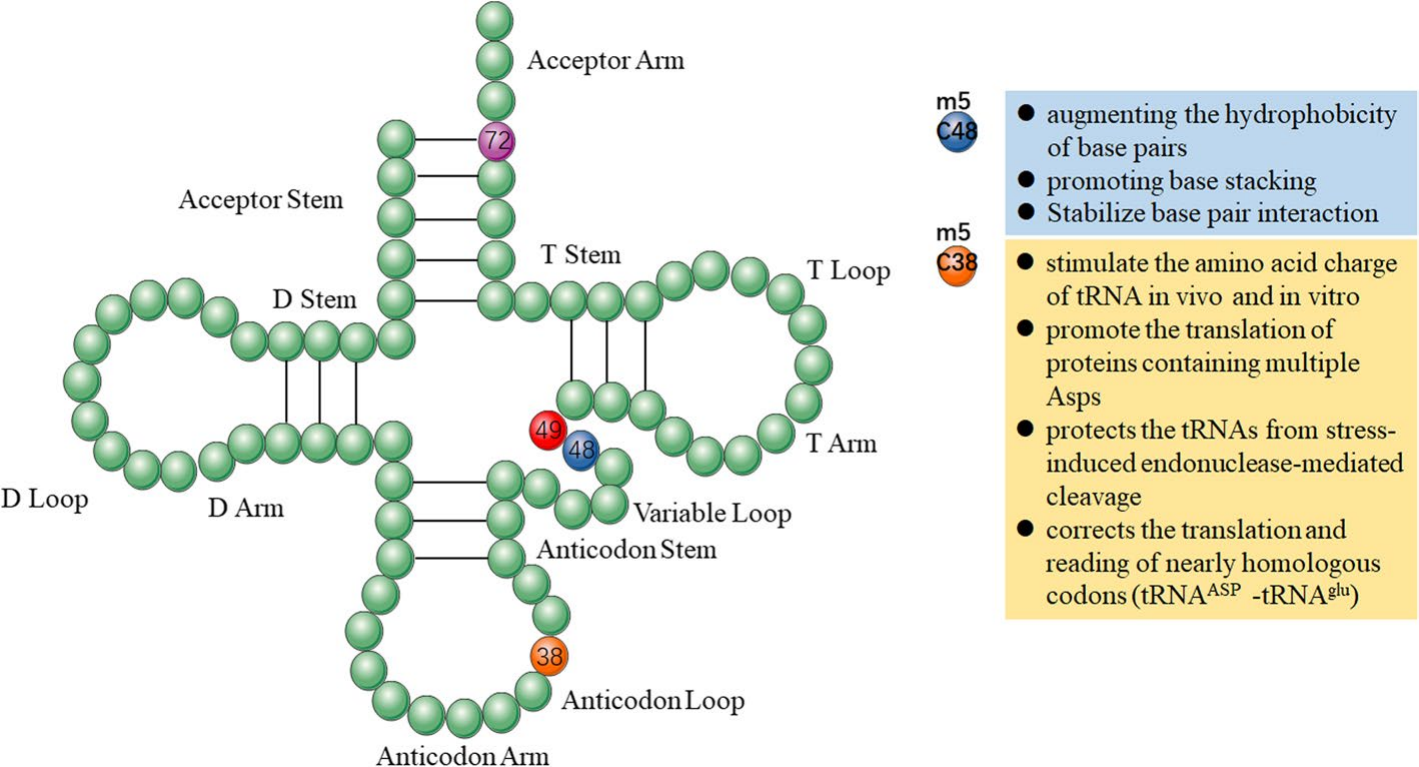
The functions of m^5^C in tRNA

Most of the researches on m^5^C modification are focused on tRNA. Methylation of tRNA most often occurs on the cytosine at the junction region between the variable ring and the T-stem, as well as on one, two, or three Cs spanning the 47-50 position [44]. They participate in the composition of secondary structure, which is in connection with the codon recognition and stability of tRNA [45]. tRNA methylation mainly affects protein synthesis in mice [46]. Some studies have shown that methylation of C48 is based on augmenting the hydrophobicity of base pairs and promoting base stacking to stabilize this interaction. An unusual “Levitt pair” is formed by C48 and the nucleoside 15 on the D ring, resulting in a characteristic L-shaped three-dimensional structure [47]. Besides C48, C38 is another frequently methylated site in the anticodon loop. Studies have confirmed that methylation of C38 in mouse tRNA^Asp^ can effectively stimulate tRNA charging Asps in vivo and in vitro, hereby promoting the translation of proteins harboring multiple Asps [48]. At the same time, C38 methylation also protects the tRNAs from stress-induced endonuclease-mediated cleavage and corrects the translation and reading of nearly homologous codons [48]. During the decoding of aspartate codons, the loss of m^5^C38 of tRNA^Met^ results in a moderated translational fidelity [49]. At the same time, DNMT2-mediated m^5^C38 tRNA^Asp^ can help to distinguish homologous and near homologous codons, such as different tRNA^ASP^ from tRNA^glu^, avoiding a false positive rate of amino acids, and improve the accuracy of translation [50]. In the late stage of tRNA^Thr^ and tRNA^Cys^ transcription, the CCA sequence is added at the 3’end and thereafter C72 was confirmed to be methylated [51]. However, there has been no specific function reported about this modification. Other mitochondrial studies have shown that mitochondrial f5C modification stems from m^5^C34 of mt-tRNA^met^, and it play a key role in mitochondrial translation as methionine in decoding unconventional AUA codons [52]. In yeast, wobble of m^5^C34 of tRNA^Leu^ is associated with translation regulation. In oxidative stress responses, its methylation level is significantly increased, which can enhance the translation of uug-codon-rich mRNAs, such as ribosomal protein El22A [47]. In addition, NSUN2-mediated tRNA m^5^C48 and m^5^C49 are located in the junction sequence of the V-loop (VL) and T-stem-loop (TSL), which is essential in accelerating tRNA stability and protein translation (Fig. 2).

In rRNA, m^5^Cs modification in the ribosome function critical region help to keep its conformation stable. In yeast, a methylated nucleotide cluster in the fourth domain of the 25 s rRNA is critical to maintain structural stability of the 60S ribosomal subunits, whereby deletion of m^5^C2278 and ribose methylation of G2288 in 25 s rRNA resulted in severe instability of the 60S ribosomal subunits [53]. The modification of m^5^C in rRNA is associated with ribosome synthesis and protein translation. Under oxidative stress, m^5^C2278 of 25S rRNA in yeast which helps to maintain rRNA folding and facilitate selective recruitment and translation of mRNAs for participation in distinct cellular signals [53]. In mitochondrial ribosomes, NSUN4 is also required for mt-m^5^C911 12S rRNA. Depletion of NSUN4 caused functional assembly defect and disrupted mitochondrial protein translation [54]. NSUN5-mediated deletion of m^5^C3782 28S rRNA hinders the synthesis of whole proteins [55].

It has been reported that if cytosine-5 methylation is absent in the vault RNA, it will be abnormally processed into Argonaute-associated small RNA fragments, which have a similar function as microRNAs [56]. Although m^5^C is necessary for stability of vault ncRNAs, m^5^C is situated in lncRNA, X-inactive specific transcripts (XIST), and it prevents Polycomb repressing complex 2 (PRC2) complexes from binding in vitro [57]. Hu et al. have investigated the distribution and regulation of lncRNA in colorectal cancer in 5hmC, and they found that the hm^5^C is distributed in lncRNA and is positively correlated with lncRNA transcription. Studies have confirmed that hm^5^C directly or indirectly regulates dysregulated colorectal cancer lncRNAs by the abnormal activity of superenhancers and promoters modified by the hm^5^C. In addition, hm^5^C also participates in long-range chromatin interactions at the lncRNA sites. They also found that lncRNAs regulated by different hm^5^C markers were associated with different clinical outcomes and tumor status [58].

As a type of RNA modification, m^5^C has relatively rich but highly dynamic characteristics, and it constitutes a multifunctional and effective mechanism for coping with constantly changing internal and external environments. It acts by regulating the intracellular RNA metabolism and related functions. m^5^C modification is increasingly becoming mainstream in the field of epitranscriptomics.

## Dynamic regulation of m^5^C by “writers”, “erasers”, and “readers”

**Fig. 3:**
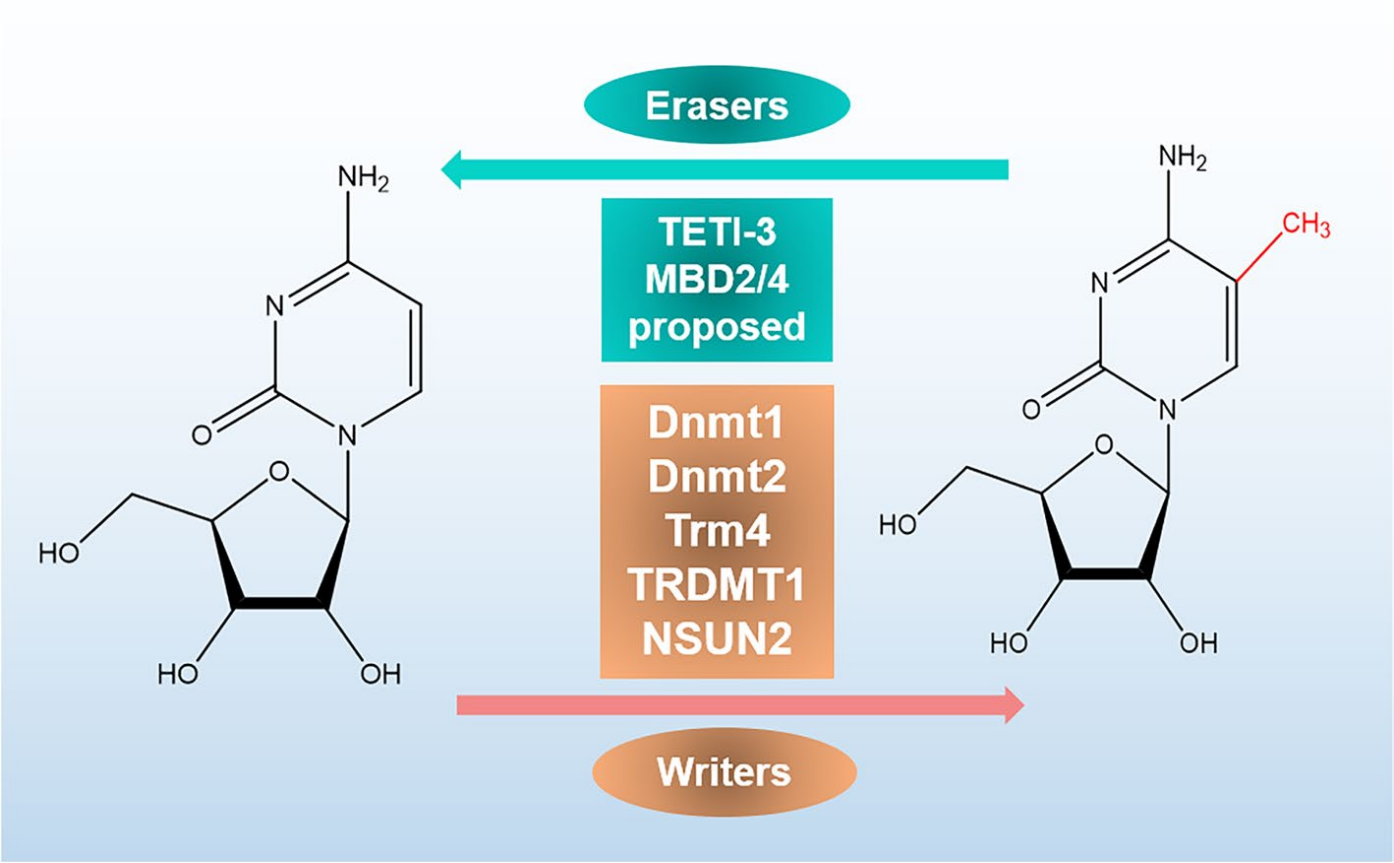
The summary of discovered writer and eraser proteins of m^5^C

m^5^C modification is mainly mediated by 3 types of proteins, namely the methyltransferases (writers), the demethylases (erasers) and the binding proteins (readers) respectively. The m^5^Cs in RNAs are recruited by NOL1/NOP2/SUN (NSUN) protein family, including NSUN1-7 and DNA methyltransferase (DNMT) homologue DNMT2 [59], NSUN1, NSUN2, and NSUN5 are infrequently expressed in eukaryotes, whereas higher eukaryotes have high expressions of NSUN3, NSUN4, NSUN6 and NSUN7 [60](Fig. 3).

## m^5^C writers

The methyltransferase of m^5^C (RCMTs) uses adenosylmethionine as a methyl donor to form m^5^C by transferring the methyl group to cytosine [61]. RCMTs are believed to be the writers for catalyzing methylation of cytosine-5, including the NSUN methyltransferases and DNMT2 [62]. More than 10 types of RNA m^5^C methyltransferases have been found, including DNMT2, and tRNA-specific methyltransferase (TRDMT) family members, NOL1/NOP2/SUN domain (NSUN) family member. Among them, the NSUN methyltransferases includes several members (NSUN1 to NSUN7) and NSUN5a/b/c [63]. The TRDMT family includes TRM4A and TRM4B in *Arabidopsis thaliana* [64]. Both NSUN and DNMT family enzymes contain conserved motif IV and motif VI [65]. DNMT2 and NSUN2 have complementary target specificities [16]. A quinte essential example in the NSUN family should be cited; cysteine at the VI position of the motif in the NSUN family interferes with C6 of the target cytosine in RNA through nucleophilic attack, meanwhile, the motif IV proline and aspartate sidechain interact with the hydrogen atom, which positions the nucleobase in the active status and form bond in transient protonation. Then, the activated nucleobase accepts the SAM methyl group. Human NSUN6 forms complex with a full-length tRNA substrate and catalyze tRNA m^5^C modification. Liu and colleagues have solved the structures of NSUN6 [66]. A non-canonical conformation of the bound tRNA can be observed from these structures, and related enzymes methylate the base portion of the target cytosine. Further biochemical analysis revealed the key, but distinction, to two conserved cysteine residues of RNA: m^5^C methylation.

## m^5^C erasers

Protein demethylases called erasers, such as the TET family of enzymes, have a reversible effect by mediating the demethylation of written RNA [67]. In recent years, quantitative analysis using LC–MS/MS/MS has shown that TET has the potential as an RNA demethylase, and its overexpression can significantly increase the level of RNA 5hmC in HEK293T cells [68]. The TET (ten-eleven translocation) proteins, TET1, TET2, and TET3, function as DNA dioxygenases catalyzing 5mC to 5hmC on DNA [69]. It has been found that oxidation of 5fC to 5caC in RNA is mediated by TET1 [70]. It is been reported that TET2 mediates the oxidation of m^5^C, which may inhibit the effect of 5-methylcytosine on the formation of double-stranded RNA in eukaryotes [71]. Besides, TET2 catalyzes RNA 5hmC, which involves degradation of RNA; this indicates that 5hmC plays a key role in post-transcriptional regulation [72]. In recent years, it has been revealed that the identified mitochondrial DNA and RNA dioxygenase Alpha-ketoglutarate-dependent dioxygenase ABH1 (ALKBH1) also participates in the demethylation of N1 methyladenosine (m^1^A) along the cytoplasmic tRNAs [73]. ALKBH1 catalyzes the anticodon modification of m^5^c34 to hm^5^cm34 (5-hydroxymethyl-20-o-methylcytidine) and f^5^cm34 (5-formyl-20-o-methylcytidine) along mt-tRNA^met^ and cytoplasmic tRNA^Leu^ [74]. Loss of ALKBH1 induce severe deficiency in mitochondrial translation and oxygen consumption, indicating that RNA m^5^C metabolism by ALKBH1 have great potential in regulating mitochondrial activity. Moreover, ALKBH1 can also specifically act on a histone dioxygenase-histone H2A [75].

## m^5^C readers

Most of the biological functions of RNA modification are related to the protein to which it binds. RNA m^5^C binding proteins, such as ALYREF and YBX1, are considered to be a type of barcode reader, which exerts biological effects by recognizing and binding to m^5^C sites [63]. Recently, m^5^C-modified RNA oligos pull-down coupled with mass spectrometry analysis has been applied to a variety of m^5^C mRNAs, and two reading proteins of m^5^C mRNA, ALYREF (RNA and export factor binding protein 2) and YBX1, have been identified [76]. ALYREF is the first mRNA reading protein identified in the nucleus and has the key m^5^C recognition site of K171. m^5^C is recognized by the m^5^C reader ALYREF through the viral RNA transcript installed in the nucleus, which helps them export to the cytoplasm [31]. In order to determine whether m^5^C installation in viral transcripts occurred in the nucleus or the cytoplasm, Eckwahl and his colleagues performed RNAs isolation from nucleus and cytoplasm separately, followed by bisulfite treatment and Sanger sequencing; they focused their functional investigations on ALYREF reads on the protein. They found that knocking out the m^5^C reading protein ALYREF has significant impact on viral protein production and viral replication. The depletion of ALYREF decreased the MLV Gag protein and virus released cells by 5-fold. On the contrary, the overexpression of ALYREF increased the Gag protein level. They also examined whether using the plasmid encoding wild- type ALYREF or the known m^5^C to recognize the impaired ALYREF mutant (K171A) could rescue the 3 T3 cells lacking endogenous ALYREF. They found that wild- type ALYREF but not mutant constructs can rescue phenotypic effects [77]. Eckwahl also confirmed that m^5^C plays an important role in bridging the interaction between ALYREF and MLV RNA through endogenous RNA immunoprecipitation (RIP). They also confirmed that ALYREF knockout decreased the cytoplasmic and nuclear ratio of m^5^C in viral RNA, due to fewer m^5^C containing viral transcripts were exported to the cytoplasm.

Unlike ALYREF, YBX1 was identified as a cytoplasmic mRNA m^5^C reading protein in human cells [78]. Based on the structural analysis of YBX1 protein and isothermal titration thermal analysis, YBX1 recognizes m^5^C in its cold shock domain through the indole ring of W65 [79]. YBX1 specifically targets several m^5^C-containing oncogenes, such as HDGF, and promotes the stability of these oncogenes and the subsequent progression of cancer by recruiting the well-known mRNA stability maintenance factor ELAVL1.

Furthermore, YBX1 could recognize about 60% of m^5^C mRNAs in urothelial bladder cancer (UCB)-derived T24 cells. In zebrafish, the maternal YBX1 loss can increase overall translation, accumulation of unfolded proteins, leading to oogenesis defects and subsequent embryogenesis failure [80]. Recent studies have also found that 87.8% of the mRNAs modified by m^5^C were identified by YBX1. Upon the early development of zebrafish embryos, 397 maternal mRNAs with m^5^C showed significantly decreased expression when YBX1 was knocked out, indicating that these m^5^Cs mediated by YBX1 played a key role in regulating maternal gene clearance during the transition from the mother to the zygote [23]. In Ypsilon Schachtel (YPS) of Drosophila, the YBX1 homologue also acted as the RNA m^5^C reading protein [81]. In Drosophila ovary, YPS promotes the homeostasis, proliferation, and differentiation of germline stem cells (GSCs), which depends on the binding of m^5^C-containing RNAs. Interestingly, like humans and zebrafish YBX1, the highly conserved cold-shock domain (CSD) also contains the YPS m^5^C binding site. Mutation of this site can induce the defective development of GSC, which indicates that RNA m^5^C modification has an effect on the development of adult stem cells [82].

**Fig. 4:**
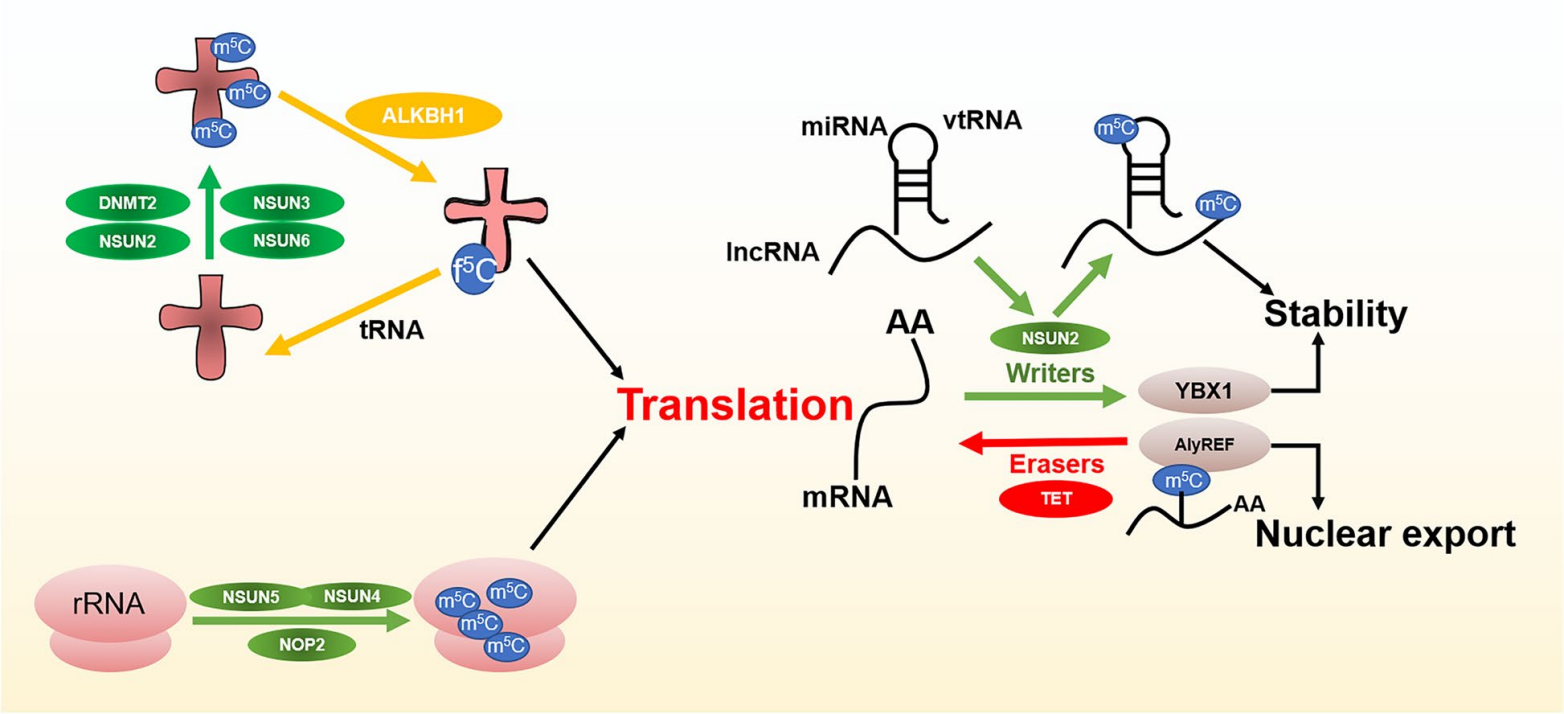
The functional landscape of m^5^C writers, readers, and erasers. m^5^C modification in tRNA or rRNA regulated by NSUN family proteins (NSUN2, NSUN3, NSUN4, NSUN5, NSUN6), NOP2, DNMT2 and ALKBH1 affects the translation. m^5^C modification of lncRNA, miRNA, vtRNA and mRNA regulated by NSUN2, YBX1, AlyREF and TET family proteins affect their stability and nuclear export

In summary, m^5^C modification extensively affects gene expression at multiple levels through interacting with a variety of writer, eraser and reader proteins (Fig. 4). Therefore, the dynamic modification of m^5^C plays multifaceted roles in various biological processes, including embryonic stem cells self-renewal and differentiation [83], circadian rhythm [84], heat shock [85] or DNA damage response [86], and sex determination [87].

## Aberrant m^5^C in cancers

**Fig. 5:**
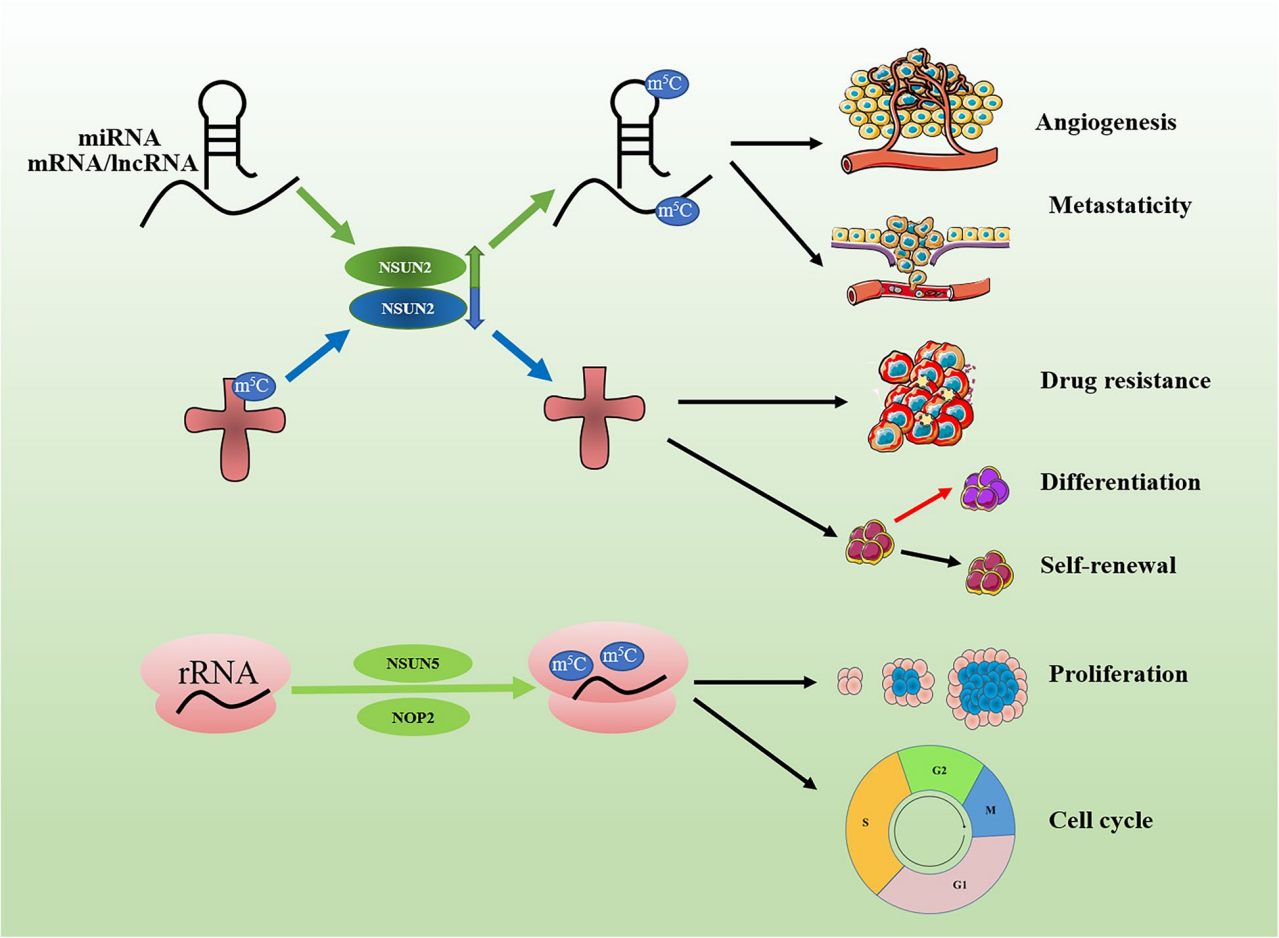
Aberrant m^5^C deposition in mRNA, miRNA and lncRNA promotes cancer angiogenesis and metastaticity. NSUN2-mediated tRNA m^5^C modification regulates cancer stem cell differentiation. m^5^C upregulation in rRNA by NOP2 or downregulation by NSUN5 disturb the cancer cell proliferation and cell cycle. Red arrows indicate induction. Blue arrows with flat end represent inhibition

It appears clear that the overall modification m^5^C and its regulators, including writers, erasers, and readers, are aberrantly expressed in various types of cancers. Emerging evidence indicates that methylation status is closely associated with the pathogenesis of cancer involving initiation, metastasis, progression, as well as drug resistance and relapse (Fig. 5).

Increased levels of RNA m^5^C can be detected in circulating tumor cells of lung cancer patients [88]. Increased gene expression of NSUN1 and NSUN2 can predict the occurrence and development of cancer [89]. Xue et al. selected m^5^C-regulated genes from the Cancer Genome Atlas (TCGA) database related to HNSCC and performed in-depth sequence analyses. They found that the expression of genes regulated by m^5^C was associated with the copy number variation (CNV) pattern. Gene set enrichment analysis (GSEA) showed that m^5^C reader protein ALYREF can actively promote mRNA export, thereby playing the role of an mRNA export adaptor in vivo and in vitro [90]. Meanwhile, DNMT1 and ALYREF expression levels effectively predicted the risk factors (morbidity and mortality) for patients with HNSCC. Similarly, based on the TCGA database, He et al. linked the abnormal changes of m^5^C regulatory genes in a variety of tumors with adverse clinical results, and provided a partial explanation for the discussion of cancer pathogenesis and/or survival. He and his colleagues assessed the correlation between the expression of m^5^C regulatory factors and related cancer pathways, and found that mutations in m^5^C regulatory genes and CNVs are significantly related in many cancer types. It is worth noting that this study verified the frequency of m^5^C regulatory patterns in 33 cancers and compared the expression levels of m^5^C regulatory factors in tumors and adjacent tissues, and found that they were overexpressed in tumors [91]. Glioma is the most common primary intracranial tumor, which is difficult to cure and often recurs. Wang et al. obtained the RNA sequence and clinicopathological data of RNA:m^5^C methyltransferase under glioma from the Chinese Glioma Genome Atlas (CGGA) and TCGA data sets. The results showed that RNA:m^5^C methyltransferase was significantly related to the malignant progression of glioma [92].

The NSUN2 gene has the highest mutation rate in gastrointestinal cancers [93]. A high frequency of mutations in m^5^C regulatory genes were found in HCC. Dysregulation of m^5^C-related genes was also associated with a higher HCC stage. Moreover, almost all patients with low survival rate were significantly associated with high expression of m^5^C regulatory factors. High expression of NSUN4 and ALYREF is highly coincident with the survival outcome. GSEA results indicate that methylation and demethylation will lead to high expression of NSUN4 [94]. In addition, the abnormal m^5^C modification of H19 lncRNA mediated by NSUN2 can affect its stability and interaction with the oncoprotein G3BP1, and it has been proved to be related to the poor differentiation of HCC. It indicates that this interaction may serve as a prognostic biomarker for HCC patients [95].

A recent study indicated that m^5^C promotes the pathogenesis of human bladder urothelial carcinoma. m^5^C stabilizes oncogene-mRNAs through the m^5^C-modified sites in their 3’ UTRs and promotes the pathogenesis of human bladder urothelial carcinoma by [31]. Zhang et al. found that NSUN2 and YBX1 is responsible for the m^5^C methylation in the 3’-untranslated region of HDGF and promote the urothelial carcinoma of the bladder. Moreover, upregulated NUSN2, YBX1, and HDGF expression levels predicted a poorer survival rate in patients with urothelial carcinoma of the bladder [96]. A recent study demonstrated the involvement of m^5^C in the development of urothelial carcinoma of the human bladder by stabilizing oncogene-mRNAs through m^5^C modified sites in their 3′ UTRs [31].

In addition, it has been shown that m^5^C and its writer proteins are associated with lineage-specific chromatin structure and treatment response/resistance, and they may be an epigenetic driver of leukemia cytochemical resistance. Studies have shown that NSUN3 and DNMT2 forms 5-azacytidine (5-AZA) sensitive chromatin structure through binding to the RNA binding protein hnRNPK, complexed with GATA1 and SPI1/PU.1, which interacts with CDK9/P-TEFb to recruit RNA polymerase-II in the nascent RNA. However, NSUN1 is recruited to the active chromatin structure formed by BRD4 and RNA polymerase-II. Though this chromatin structure is insensitive to 5-AZA, it is highly sensitive to the BRD4 inhibitors JQ1 and NSUN1 knockdown. Furthermore, RNA m^5^C and NSUN1−/BRD4 related active chromatin were enriched in 5-AZA-resistant leukemia cells and 5-AZA-resistant myelodysplastic syndrome and acute myeloid leukemia specimens [97].

**Fig. 6:**
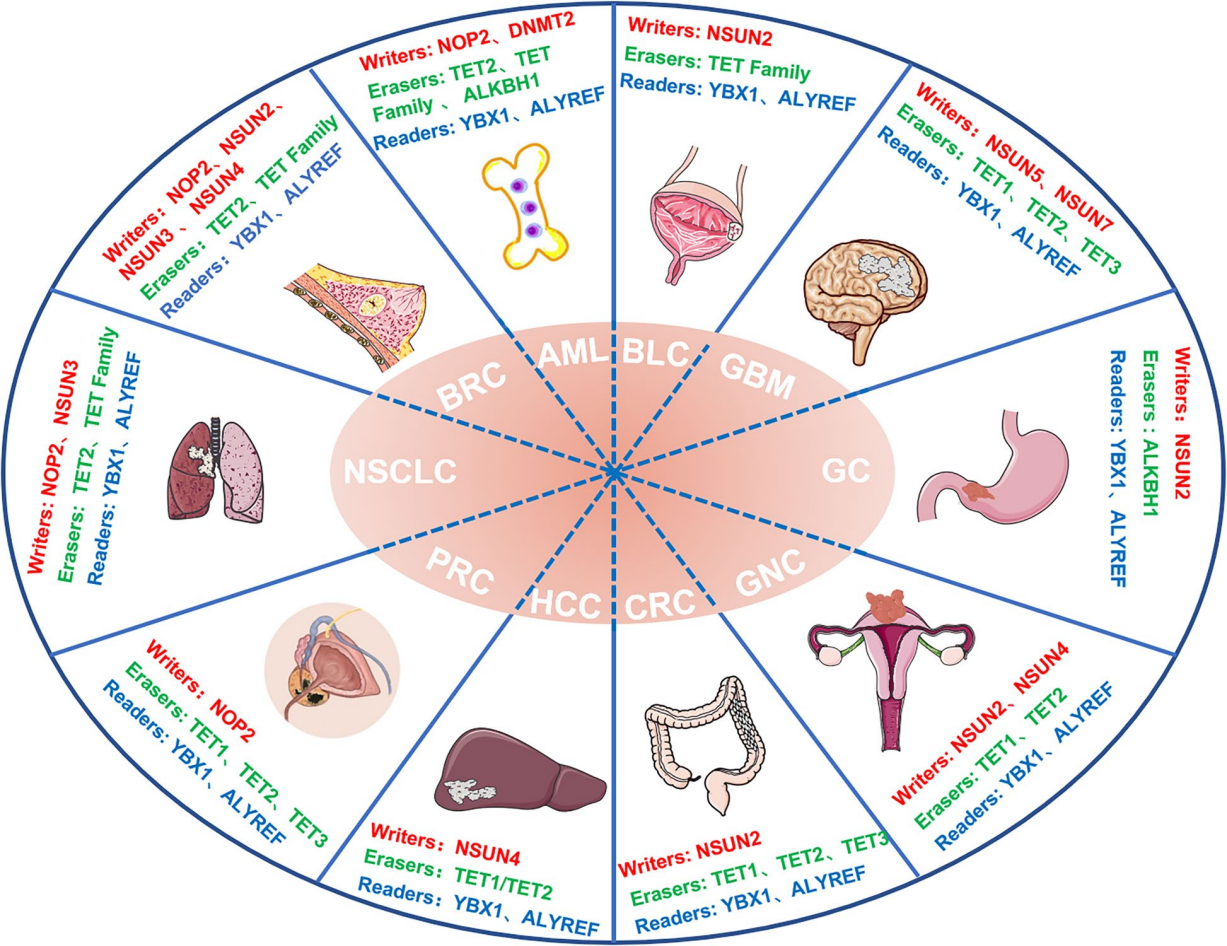
Role of aberrant expressions of m^5^C writer, reader and eraser proteins in various cancer types. NSCLC: Non-Small Cell Lung Cancer; BRC: Breast Cancer. AML: Acute myeloid leukemia; BLC: Bladder Cancer; GBM: Glioblastoma; GC: Gastric Cancer; GNC: Gynecologic Cancer; CRC: Colorectal Cancer; HCC: Hepatocellular carcinoma; PRC: Prostate Cancer; HDFs: Human diploid fibroblasts; U87: Human glioma cell line U87; ATX: Autotaxin

In summary, aberrant expressions of m^5^C writer, eraser and reader proteins are found in various human cancers (Fig. 6, Table 1).

**Table 1:** The role and mechanism of m^5^C in cancer

m^5^C regulators	Position	Function	Potential mechanism	Refs
ALYREF, NSUN5, DNMT1	HNSCC	Participate in progress and predict prognosis	Involved in regulating tumor energy metabolism and protein synthesis	[90]
NSUN2	Gastric Cancer	Cell Proliferation	Regulate downstream gene p57Kip2	[98]
NSUN2	HDFs	Cell Proliferation and Senescence	Regulate the expression of p27 and CDK1	[99]
NSUN2	U87	Cell Migration	Participate in regulation by methylating ATX mRNA	[100]
NSUN2	HCC	Cell Differentiation	Recruit oncoprotein G3BP1	[95]
NSUN2, ALYREF	Bladder Cancer	Cell Proliferation and Migration	Regulation of mRNA stability through a new binding protein YBX1 in the cytoplasm	[31]
NSUN6	Pancreatic Cancer	Cell Proliferation	Regulates cell cycle and G2M checkpoints	[101]
NSUN4, ALYREF	HCC	Tumor progression	High expression of NSUN4 and ALYREF is significantly correlated with survival outcome	[94]
NSUN2, NSUN6	TNBC	Tumor proliferation Occurrence and Metastasis.	Affect tumor development and tumor immune microenvironment	[102]
NSUN2	Gastrointestinal Cancer	Tumor progression and pathological staging	Regulates GSK3B and participates in the ErbB/PI3K-Akt signaling pathway	[103]
DNMT3A	Glioblastoma Multiforme	Tumor progression	Inhibit the formation of miRNA/mRNA duplexes, loss of tumor suppression kinetic energy	[104]
ALYREF	Bladder Cancer	Glycolysis and Tumorigenesis	Overexpression of ALYREF promotes the proliferation of bladder cancer cells through PKM2-mediated glycolysis.	[105]

## m^5^C and Cancer immunotherapy

**Fig. 7:**
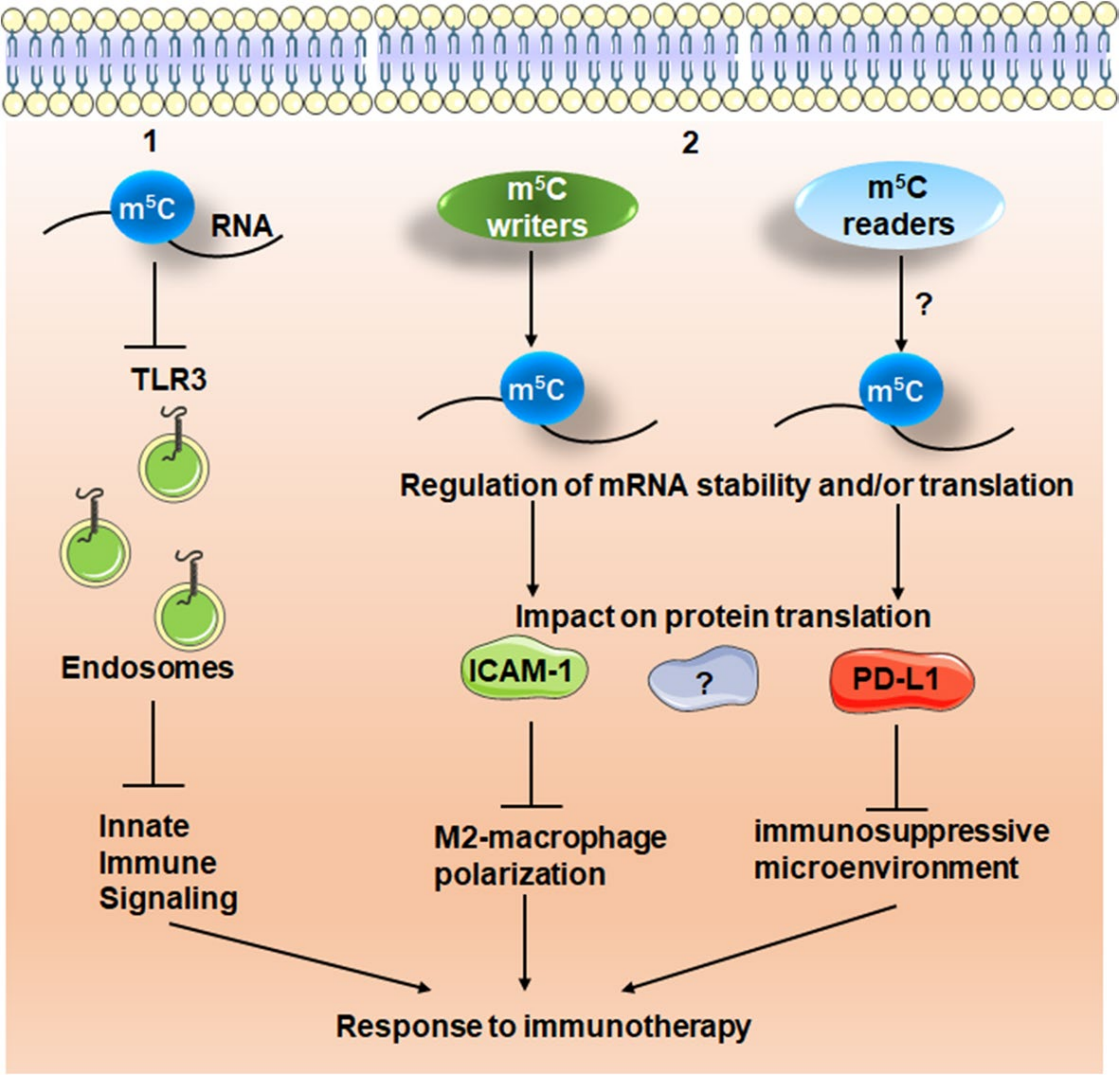
The identified functions of m^5^C in regulation of immune system

The concept of protein replacement therapy using a gene was first proposed 30 years ago [106]. However, due to the fact that RNA molecule is instable and can induce immunogenicity, this approach did not gain propagation. Later, Kariko group incorporated m^5^C into mRNA therapy either separately or in combination, and it reduced the toll-like receptor (TLR)- mediated immune effects of mRNA [9]. This marks the breakthrough in the RNA and immunotherapy field that base modification in mRNA alters the immune activity. Andries et al. [107] believed that m^5^C-mRNA can be used as a new approach in the field of mRNA-based therapeutics, partially due to that m^5^C-modified mRNA may evade the innate immune system through inhibition of endosomal Toll-like receptor 3(TLR3) (Fig. 7).

## m^5^C implications in tumor immune microenvironment

Increasing evidence based on bioinformatic analysis implicate the significance of m^5^C in tumor immune microenvironment. Diverse methylation regulators can be used as prognostic and diagnostic markers of cancer [108, 109]. Oral Squamous Cell Carcinoma (OSCC) patients with higher expression levels of m^5^C regulators had lower immune activity in TILs such as DCs, NK cells, and CD8+ T-cells [110]. Papillary thyroid cancer (PTC) patients with low m^5^C-score contained higher resting CD4+ memory T cells and CD8+ T cells and had better prognosis, while activated NK cells and monocytes were mostly enriched in the high m^5^C-score patients with worse prognosis [111]. In triple-negative breast cancer, the changes in the expression of m^5^C RNA methylation regulators, the up-regulation of NSUN2 expression and the down-regulation of NSUN6 expression, which can objectively predict the clinical prognostic risk of TNBC patients to a large extent. Therefore, it may become new prognostic markers of TNBC and provide clues for understanding the RNA epigenetic modification of TNBC [102]. Related studies have also confirmed that NSUN3 and NSUN4 can predict the prognosis of lung squamous cell carcinoma and regulate the immune microenvironment [112]. In lung adenocarcinoma patients, different m^5^C pattern had different TME immune cell infiltration and high m^5^C score group had a better prognosis [113]. Besides, m^5^C-regulated lncRNAs also predict the overall survival of lung adenocarcinoma patients and affect the tumor immune microenvironment [114]. In pancreatic cancer patients, 3 m^5^C-related lncRNAs showed prognostic value. With TIDE (Tumor Immune Dysfunction and Exclusion) algorithm, high-m^5^C-lncRNA scores had a better response to immunotherapy [115]. In another study of pancreatic ductal adenocarcinoma (PDAC), relationships between m^5^C-related lncRNAs and PDAC-infiltrating immune cells were evaluated. Naïve B cells, CD8 + T cells, Treg cells, and resting NK cells had a higher expression level in the low-risk group while the M0 and M2 phenotype macrophages had a higher expression level in the high-risk group, suggesting that m^5^C-related lncRNAs may regulate pancreatic cancer progression via promoting M2 phenotype macrophage polarization or infiltration in PDAC [116]. Related expression also exists in the research of Head and Neck Squamous Cell Carcinoma [117], hepatocellular carcinoma [118], breast cancer [119], human high-grade serous ovarian cancer [120], cutaneous melanoma [121] and colon cancer [122].

## m^5^C writers in regulation of cancer immunotherapy

Immune escape is a hallmark of human cancer. T-cell exhaustion mainly accounts for escape of tumors from immune surveillance. NSUN2, a tRNA methyltransferase, modifies tRNA and mRNA by increasing the methylation of m^5^C. NSUN2 is an important factor in maintaining stem cell self-renewal and differentiation [123]. In head and neck squamous cell carcinoma (HNSCC), the combination of NSUN2 expression and T-cell activation correlates with patient survival regardless of the HPV status, indicating that NSUN2 may serve as a potential biomarker for immune-checkpoint blockade [124]. It has been reported that NSUN2 methylates ICAM-1 (Intercellular Adhesion Molecule 1) mRNA and promotes its translation [29], thus inhibiting M2-macrophage polarization and suppressing tumor metastasis (Fig. 7). Moreover, targeting NSUN2 expression may also improve the outcome of immunotherapy in HNSCC [125]. Nonetheless, a larger sample size is necessary to further validate how the NSUN2 affects immune checkpoint blockade outcome [126].

## m^5^C readers in regulation of cancer immunotherapy

YBX1 protein is the most common tumor-associated antigen and can also induce T cell response [127]. Therefore it can be used as a target of effector immunity and a candidate vaccine for evaluation [128]. Chemotherapy induces an immunosuppressive microenvironment in tumors and induces immune evasion through YBX1-mediated upregulation of PD-L1 (programmed death-1 ligand 1) (Fig. 7). YBX1 is upregulated in chemoresistant HCC cells. YBX1 knockout reverses chemoresistance by blocking PD-L1 expression and activating T cells in a tumor microenvironment [129]. Upregulation of functional cytotoxic CD8^+^ T cells and downregulation of myeloid-derived suppressor cells and regulatory T cells are associated with the overcome of the tumor immunosuppressive environment and immune escape status. YBX1 drives signal transduction in a tumor immunosuppressive microenvironment and immune escape pathway. In addition, YBX1 knockout can reverse HCC drug resistance by blocking PD-L1 expression and activating T cells in a tumor microenvironment. Data presented by Tao et al. showed that both tumor immune evasion and multidrug resistance can be reversed by targeting the YBX1 signaling cascade, suggesting an effective treatment regimen against tumor chemoresistance [130].

In conclusion, these data implicate that modulation of m^5^C regulators could be a useful strategy.

## Conclusions and perspectives

m^5^C modifications of RNA have been demonstrated as a new molecular mechanism that controls eukaryotes gene expression. How the modification of m^5^C affects the ncRNA biogenesis, localization, and function, and how these effects are associated with etiology of cancer are to be elucidated. In recent years, extensive efforts have been devoted to the study of m^5^C modifications in cancer. In this review, particular attention was focused to Writer-NSUN2, the Eraser-TET family, and Reader-YBX1..

Some proof-of-concept studies have revealed that maladjusted m^5^C modulators targeted by small-molecule inhibitors have therapeutic potential for cancer treatment. As of now, specific m^5^C inhibitors have not been developed. In DNA methylation, the chemicals initially developed to target these proteins, such as 5-aza-2-deoxycytidine (decitabine) or 5-azacytidine (Vidaza), have been approved to treat myelodysplastic syndrome by the FDA, but their usage in solid tumor treatments is limited.

Bioinformatic research in RNA m^5^C modification will also be critical in both identifying the m^5^C sites from Single-molecule sequencing and in prediction of m^5^C sites in different RNA species [131]. Several machine-learning algorithms have recently been developed to predict the m^5^C sites in *Homo sapiens*, *Mus musculus* and *Arabidopsis thaliana* [34, 132]. With the fast development of computational approaches, it can be expected that the m^5^C epitranscriptomics will be fully understood and its relation with cancer will be uncovered.

In the future, the detailed roles of RNA m^5^C in immune system regulation and tumor immune microenvironment will be important directions. As for RNA m^6^A, depletion of m^6^A writers, readers or erasers had significant phenotypic consequences in the cellular response to infection and in a few immune cell types. Whether RNA m^5^C exerts similar functions in the immune system deserves deep efforts. More importantly, how different RNA modifications combat or combine with each other in immune regulation needs a long way to explore.

Different from m^6^A, the RNA m^5^C modification partially shares the same enzymatic machinery including DNMT1 with DNA m^5^C. This mechanism may also play critical roles during the tumor development and progression. m^5^C can operate with the tumor cells and immune microenvironment to brake or accelerate the tumorigenesis. It will be interesting to identify the immune checkpoint molecular target of m^5^C in the tumor microenvironment, so that targeted therapy can be used in combination with checkpoint immunotherapy to better treat cancer. In general, the investigation of novel m^5^C epigenetic modification in cancer not only will provide new insights into the molecular mechanisms of cancer biology and immune response, but also will pave the way to the develop new promising therapies.

## Data Availability

The authors confirm that the data support the findings of this study are available within the article.

## Abbreviations

m^6^A: N6-methyladenosine
m^5^C: 5-methylcytosine
UTRs: Untranslated regions
tRNA: Transfer RNA
rRNA: Ribosomal RNA
mRNA: Messenger RNA
snRNA: Small nuclear RNA
miRNA: Micro RNA
lncRNA: Long non-coding RNA
eRNA: Enhancer RNA
5hmC: 5-hydroxymethylcytosine
5fC: 5-formylcytosine
5caC: 5-carboxycytosine
DNMT: DNA methyltransferase
LC-MS: Liquid chromatography-mass spectrometry
CDS: Coding sequence
RCMTs: RNA:m^5^C methyltransferases
TRDMT: TRNA-specific methyltransferase
TET: Ten-eleven translocation
ALKBH1: Alpha-ketoglutarate-dependent dioxygenase ABH1
hm5cm34: 5-hydroxymethyl-20-o-methylcytidine
f5cm34: 5-formyl-20-o-methylcytidine
RIP: RNA immunoprecipitation
GSCs: Germline stem cells
CSD: Cold-shock domain
VL: V-loop
TSL: T-stem and loop
XIST: X-inactive specific transcripts
PRC2: Polycomb repressing complex 2
CNV: Copy number variation
GSEA: Enrichment analysis
TFs: Transcription factors
5-AZA: 5-azacytidine
FDA: Food and Drug Administration
MDS: Myelodysplastic syndrome
AML: Acute myelogenous leukemia
SGC: Structural Genome Consortium
SAM: S-adenosylmethionine
5’FU: 5-fluorouracil
GSC: Germline stem cell
S1P: Phingosine-1-phosphate
SPHK: Sphingosine kinase
BC: Breast cancer
PCa: Prostate cancer
TLR: Toll-like receptor
SLE: Systemic lupus erythematosus
CR: Coding region
HNSCC: Head and neck squamous cell carcinoma
CTLA4: Cytotoxic T lymphocyte antigen 4
PD-1: Programmed cell death 1
ICAM-1: Intercellular adhesion molecule 1
PD-L1: Programmed death-1 ligand 1
